# Supplementary material for: E3 Ubiquitin Ligase TRIP12: Regulation, Structure, and Physiopathological Functions
Source: Int J Mol Sci. 2020 Nov 12;21(22):8515. doi: 10.3390/ijms21228515 (PMC7697007; doi:10.3390/ijms21228515)
Supplement: Supplementary file 1 [file ijms-21-08515-s001.pdf]

Supplemental Table. The Thyroid hormone Receptor Interacting Proteins family and their functional roles are presented except for TRIP12, which is the topic of this review.

	Uniprot Human	Alternative Short Name	Alternative Name	Function
**TRIP1**	P62195	PRS8 P45/SUG1	26S proteasome regulatory subunit 8, 26S proteasome AAA-ATPase subunit RPT6, Proteasome 26S subunit ATPase 5. Proteasome subunit p45	Transcriptional activator [1–4] p45 ATPase subunit of PA700, the regulatory complex of the 26S proteasome [5,6].
**TRIP2**	Q15648	MED1	Mediator of RNA polymerase II transcription subunit 1	Component of the Mediator complex, a coactivator involved in the regulated transcription of nearly all RNA polymerase II-dependent genes [7].
		TRAP220	Thyroid hormone receptor-associated protein complex 220 kDa component	Interaction with nuclear receptors in a ligand-dependent manner, global activator for the nuclear receptor superfamily [8–10].
		DRIP205	Vitamin D receptor-interacting protein complex component	
		ARC205	Activator-recruited cofactor 205 kDa component	
		CRSP200	Cofactor Required for Sp1	Subunit p200 of the transcriptional factor CRSP is identical to TRIP2 [11].
		PBP, PPAR-binding protein	Peroxisome proliferator-activated receptor-binding protein	Interaction with peroxisome proliferator-activated receptor [12].
		RB18A	Recognized by PAb1801 antibody	Identification of common antigenic and functional properties with P53 [13].
**TRIP3**	Q15649	ZNHIT3	ZNHI3 Zinc finger HIT domain-containing protein 3	The heterodimer NUFIP1 (nuclear fragile X mental retardation-interacting protein 1)-ZNHIT3 is implicated in the assembly of the box C/D small nucleolar ribonucleoprotein (snoRNP) [14] and in starvation-induced ribophagy [15].
		HNF-4a coactivator	Hepatocyte Nuclear Factor 4a coactivator	[16]
**TRIP4**	Q15650	ASC-1	Activating signal cointegrator 1	Transcription coactivator that associates with nuclear receptors, transcriptional coactivators and basal transcription factors [17]. Component of the human RQC-trigger (hRQT) complex that facilitates ribosome-associated quality control [18].
**TRIP5**	P52732	KIF11 HKSP Eg5	Kinesin-like protein KIF11 Kinesin-like spindle protein	Motor protein required for establishing a bipolar spindle during mitosis [19,20]. Required in non-mitotic cells for transport of secretory proteins from the Golgi complex to the cell surface [21]. Modulates neuronal migration [21,22].
**TRIP6**	Q15654	ZRP-1 OIP-1	Zyxin-related protein 1 Opa-interacting protein 1	Relays signals from the cell surface to the nucleus to weaken adherent junction and promote actin cytoskeleton reorganization and

				cell invasiveness, transcriptional coactivator for NF-kappa-B and JUN [23].
**TRIP7**	Q15651	HMGN3	High mobility group nucleosome-binding domain-containing protein 3	Binds to nucleosomes, regulating chromatin structure [24,25].
**TRIP8**	Q15652	JMJD1C	Probable JmjC domain-containing histone demethylation protein 2C	Probable histone demethylase that specifically demethylates Lysine 9 residue of histone H3 [26].
**TRIP9**	Q15653	NF-Kappa-BIB	NF-kappa-B inhibitor beta	Inhibits NF-kappa-B by complexing with and trapping it in the cytoplasm [27].
**TRIP10**	Q15642	CIP4<br>STP	Cdc42-interacting protein 4<br>Salt tolerant protein<br>Protein Felic	Required for translocation of GLUT4 to the plasma membrane [28]. Required for membrane tubulation during endocytosis, the formation of podosomes. Promotes membrane invagination and the formation of tubules. Promotes CDC42-induced actin polymerization [29].
**TRIP11**	Q15643	GMAP-210<br>CEV14	Golgi-associated microtubule-binding protein 210<br>Clonal evolution-related gene on chromosome 14 protein	Is a membrane tether required for vesicle tethering to Golgi, essential role in the maintenance of Golgi structure and function [30].
**TRIP12**	Q14669			
**TRIP13**	Q15645	PCH2<br>16E1-BP	Pachytene checkpoint protein 2 homolog<br>Human papillomavirus type 16 E1 protein-binding protein	Plays a key role in chromosome recombination and chromosome structure development during meiosis [31]. Promotes early steps of the DNA double-strand breaks (DSBs) repair process [32]. Plays a role in mitotic spindle assembly checkpoint activation [33].
**TRIP14**	Q15646	OASL<br>2′-5′-OAS-RP<br>p59OASL	2′-5′-oligoadenylate synthase-like protein<br>2′-5′-OAS-related protein<br>p59OASL 59 kDa 2′-5′-oligoadenylate synthase-like protein	Can bind double-stranded RNA. Displays antiviral activity against encephalomyocarditis and hepatitis C virus [34].
**TRIP15**	P61201	SGN2	COP9 signalosome complex subunit 2<br>Alien homolog<br>JAB1-containing signalosome subunit 2	Essential component of the COP9 signalosome complex (CSN) [35].

References

1. Baur, E.V.; Zechel, C.; Heery, D.; Heine, M.J.; Garnier, J.M.; Vivat, V.; Le Douarin, B.; Gronemeyer, H.; Chambon, P.; Losson, R. Differential ligand-dependent interactions between the AF-2 activating domain of nuclear receptors and the putative transcriptional intermediary factors mSUG1 and TIF1. *EMBO J.* **1996**, *15*, 110–124, doi:10.1002/j.1460-2075.1996.tb00339.x.
2. Swaffield, J.C.; Melcher, K.; Johnston, S.A. A highly conserved ATPase protein as a mediator between acidic activation domains and the TATA-binding protein. *Nat. Cell Biol.* **1995**, *374*, 88–91, doi:10.1038/374088a0.
3. Lee, J.W.; Ryan, F.; Swaffield, J.C.; Johnston, S.A.; Moore, D.D. Interaction of thyroid-hormone receptor with a conserved transcriptional mediator. *Nat. Cell Biol.* **1995**, *374*, 91–94, doi:10.1038/374091a0.
4. Ingley, E.; Chappell, D.; Poon, S.Y.K.; Sarna, M.K.; Beaumont, J.G.; Williams, J.H.; Stillitano, J.P.; Tsai, S.; Leedman, P.J.; Tilbrook, P.A.; et al. Thyroid Hormone Receptor-interacting Protein 1 Modulates Cytokine and

Nuclear Hormone Signaling in Erythroid Cells. *J. Biol. Chem.* **2001**, *276*, 43428–43434, doi:10.1074/jbc.m106645200.

5. Su, K.; Yang, X.; Roos, M.D.; Paterson, A.J.; Kudlow, J.E. Human Sug1/p45 is involved in the proteasome-dependent degradation of Sp1. *Biochem. J.* **2000**, *348*, 281–289.
6. Yamada, H.Y.; Gorbsky, G.J. Inhibition of TRIP1/S8/hSug1, a component of the human 19S proteasome, enhances mitotic apoptosis induced by spindle poisons. *Mol. Cancer Ther.* **2006**, *5*, 29–38, doi:10.1158/1535-7163.mct-05-0126.
7. Fondell, J.D.; Brunel, F.; Hisatake, K.; Roeder, R.G. Unliganded thyroid hormone receptor alpha can target TATA-binding protein for transcriptional repression. *Mol. Cell. Biol.* **1996**, *16*, 281–287, doi:10.1128/mcb.16.1.281.
8. Kang, Y.K.; Guermah, M.; Yuan, C.-X.; Roeder, R.G. The TRAP/Mediator coactivator complex interacts directly with estrogen receptors alpha and beta through the TRAP220 subunit and directly enhances estrogen receptor function in vitro. *Proc. Natl. Acad. Sci. USA* **2002**, *99*, 2642–2647.
9. Rachez, C.; Lemon, B.D.; Suldan, Z.; Bromleigh, V.; Gamble, M.J.; Näär, A.M.; Erdjument-Bromage, H.; Tempst, P.; Freedman, L.P. Ligand-dependent transcription activation by nuclear receptors requires the DRIP complex. *Nat. Cell Biol.* **1999**, *398*, 824–828, doi:10.1038/19783.
10. Yuan, C.-X.; Ito, M.; Fondell, J.D.; Fu, Z.-Y.; Roeder, R.G. The TRAP220 component of a thyroid hormone receptor- associated protein (TRAP) coactivator complex interacts directly with nuclear receptors in a ligand-dependent fashion. *Proc. Natl. Acad. Sci. USA* **1998**, *95*, 7939–7944, doi:10.1073/pnas.95.14.7939.
11. Ryu, S.; Zhou, S.; Ladurner, A.G.; Tjian, R. The transcriptional cofactor complex CRSP is required for activity of the enhancer-binding protein Sp1. *Nat. Cell Biol.* **1999**, *397*, 446–450, doi:10.1038/17141.
12. Zhu, Y.; Qi, C.; Jain, S.; Rao, M.S.; Reddy, J.K. Isolation and Characterization of PBP, a Protein That Interacts with Peroxisome Proliferator-activated Receptor. *J. Biol. Chem.* **1997**, *272*, 25500–25506, doi:10.1074/jbc.272.41.25500.
13. Drané, P.; Barel, M.; Balbo, M.; Frade, R. Identification of RB18A, a 205 kDa new p53 regulatory protein which shares antigenic and functional properties with p53. *Oncogene* **1997**, *15*, 3013–3024, doi:10.1038/sj.onc.1201492.
14. Quinternet, M.; Rothé, B.; Barbier, M.; Bobo, C.; Saliou, J.-M.; Jacquemin, C.; Back, R.; Chagot, M.-E.; Cianferani, S.; Meyer, P.; et al. Structure/Function Analysis of Protein–Protein Interactions Developed by the Yeast Pih1 Platform Protein and Its Partners in Box C/D snoRNP Assembly. *J. Mol. Biol.* **2015**, *427*, 2816–2839, doi:10.1016/j.jmb.2015.07.012.
15. Wyant, G.A.; Abu-Remaileh, M.; Frenkel, E.M.; Laqtom, N.N.; Dharamdasani, V.; Lewis, C.A.; Chan, S.H.; Heinze, I.; Ori, A.; Sabatini, D.M. NUFIP1 is a ribosome receptor for starvation-induced ribophagy. *Sci.* **2018**, *360*, 751–758, doi:10.1126/science.aar2663.
16. Iwahashi, H.; Yamagata, K.; Yoshiuchi, I.; Terasaki, J.; Yang, Q.; Fukui, K.; Ihara, A.; Zhu, Q.; Asakura, T.; Cao, Y.; et al. Thyroid Hormone Receptor Interacting Protein 3 (Trip3) Is a Novel Coactivator of Hepatocyte Nuclear Factor-4. *Diabetes* **2002**, *51*, 910–914, doi:10.2337/diabetes.51.4.910.
17. Kim, H.-J.; Yi, J.-Y.; Sung, H.-S.; Moore, D.D.; Jhun, B.H.; Lee, Y.C.; Lee, J.W. Activating Signal Cointegrator 1, a Novel Transcription Coactivator of Nuclear Receptors, and Its Cytosolic Localization under Conditions of Serum Deprivation. *Mol. Cell. Biol.* **1999**, *19*, 6323–6332, doi:10.1128/mcb.19.9.6323.
18. Hashimoto, S.; Sugiyama, T.; Yamazaki, R.; Nobuta, R.; Inada, T. Identification of a novel trigger complex that facilitates ribosome-associated quality control in mammalian cells. *Sci. Rep.* **2020**, *10*, 3422, doi:10.1038/s41598-020-60241-w.
19. Sawin, K.E.; Mitchison, T.J. Mutations in the kinesin-like protein Eg5 disrupting localization to the mitotic spindle. *Proc. Natl. Acad. Sci. USA* **1995**, *92*, 4289–4293, doi:10.1073/pnas.92.10.4289.
20. Hata, S.; Peidro, A.P.; Panic, M.; Liu, P.; Atorino, E.; Funaya, C.; Jäkle, U.; Pereira, G.; Schiebel, E. The balance between KIFC3 and EG5 tetrameric kinesins controls the onset of mitotic spindle assembly. *Nat. Cell Biol.* **2019**, *21*, 1138–1151, doi:10.1038/s41556-019-0382-6.
21. Wakana, Y.; Villeneuve, J.; van Galen, J.; Cruz-Garcia, D.; Tagaya, M.; Malhotra, V. Kinesin-5/Eg5 is important for transport of CARTS from the trans-Golgi network to the cell surface. *J. Cell Biol.* **2013**, *202*, 241–250.
22. Falnikar, A.; Tole, S.; Baas, P.W. Kinesin-5, a mitotic microtubule-associated motor protein, modulates neuronal migration. *Mol. Biol. Cell* **2011**, *22*, 1561–1574, doi:10.1091/mbc.e10-11-0905.
23. Willier, S.; Butt, E.; Richter, G.H.; Burdach, S.; Grunewald, T.G.P. Defining the role of TRIP6 in cell physiology and cancer. *Biol. Cell* **2011**, *103*, 573–591, doi:10.1042/bc20110077.

24. West, K.L.; Ito, Y.; Birger, Y.; Postnikov, Y.; Shirakawa, H.; Bustin, M. HMGN3a and HMGN3b, Two Protein Isoforms with a Tissue-specific Expression Pattern, Expand the Cellular Repertoire of Nucleosome-binding Proteins. *J. Biol. Chem.* **2001**, *276*, 25959–25969, doi:10.1074/jbc.m101692200.
25. Barkess, G.; Postnikov, Y.; Campos, C.D.; Mishra, S.; Mohan, G.; Verma, S.; Bustin, M.; West, K. The chromatin-binding protein HMGN3 stimulates histone acetylation and transcription across the Glyt1 gene. *Biochem. J.* **2012**, *442*, 495–505, doi:10.1042/bj20111502.
26. Katoh, M.; Katoh, M. Comparative integromics on JMJD1C gene encoding histone demethylase: Conserved POU5F1 binding site elucidating mechanism of JMJD1C expression in undifferentiated ES cells and diffuse-type gastric cancer. *Int. J. Oncol.* **2007**, *31*, 219–223.
27. Baldwin, A.S. The NF-kappa B and I kappa B proteins: New discoveries and insights. *Annu. Rev. Immunol.* **1996**, *14*, 649–683.
28. Hartig, S.M.; Ishikura, S.; Hicklen, R.S.; Feng, Y.; Blanchard, E.G.; Voelker, K.A.; Pichot, C.S.; Grange, R.W.; Raphael, R.M.; Klip, A.; et al. The F-BAR protein CIP4 promotes GLUT4 endocytosis through bidirectional interactions with N-WASp and Dynamin-2. *J. Cell Sci.* **2009**, *122*, 2283–2291, doi:10.1242/jcs.041343.
29. Itoh, T.; Erdmann, K.S.; Roux, A.; Habermann, B.; Werner, H.; De Camilli, P. Dynamin and the Actin Cytoskeleton Cooperatively Regulate Plasma Membrane Invagination by BAR and F-BAR Proteins. *Dev. Cell* **2005**, *9*, 791–804, doi:10.1016/j.devcel.2005.11.005.
30. Zucchetti, A.E.; Bataille, L.; Carpier, J.-M.; Dogniaux, S.; Roman-Jouve, M.S.; Maurin, M.; Stuck, M.W.; Rios, R.M.; Baldari, C.T.; Pazour, G.J.; et al. Tethering of vesicles to the Golgi by GMAP210 controls LAT delivery to the immune synapse. *Nat. Commun.* **2019**, *10*, 1–17, doi:10.1038/s41467-019-10891-w.
31. Roig, I.; Dowdle, J.A.; Toth, A.; de Rooij, D.G.; Jasin, M.; Keeney, S. Mouse TRIP13/PCH2 is required for recombination and normal higher-order chromosome structure during meiosis. *PLoS Genet.* **2010**, 6, e1001062.
32. Clairmont, C.S.; Sarangi, P.; Ponnienselvan, K.; Galli, L.D.; Csete, I.; Moreau, L.; Adelmant, G.; Chowdhury, D.; Marto, J.A.; D'Andrea, A.D. TRIP13 regulates DNA repair pathway choice through REV7 conformational change. *Nat. Cell Biol.* **2020**, *22*, 87–96, doi:10.1038/s41556-019-0442-y.
33. Défachelles, L.; Russo, A.E.; Nelson, C.R.; Bhalla, N. The conserved AAA-ATPase PCH-2 TRIP13 regulates spindle checkpoint strength. *Mol. Biol. Cell* **2020**, *31*, 2219–2233, doi:10.1091/mbc.e20-05-0310.
34. Ishibashi, M.; Wakita, T.; Esumi, M. 2′,5′-Oligoadenylate synthetase-like gene highly induced by hepatitis C virus infection in human liver is inhibitory to viral replication in vitro. *Biochem. Biophys. Res. Commun.* **2010**, *392*, 397–402, doi:10.1016/j.bbrc.2010.01.034.
35. Schaefer, L.; Beermann, M.L.; Miller, J.B. Coding Sequence, Genomic Organization, Chromosomal Localization, and Expression Pattern of the Signalosome ComponentCops2: The Mouse Homologue of Drosophila Alien. *Genomics* **1999**, *56*, 310–316, doi:10.1006/geno.1998.5728.
